# Changes in the Risk of Stroke in Dialysis Patients: A Retrospective Analysis over the Last 40 Years

**DOI:** 10.3390/toxins13050350

**Published:** 2021-05-13

**Authors:** Toshiya Aono, Yuki Shinya, Satoru Miyawaki, Takehiro Sugiyama, Isao Kumagai, Atsumi Takenobu, Masahiro Shin, Nobuhito Saito, Akira Teraoka

**Affiliations:** 1 Department of Neurosurgery, Teraoka Memorial Hospital, Hiroshima 729-3103, Japan; t.aono0429@gmail.com (T.A.); ikyoku@teraoka-hosp.jp (A.T.); shintmh@teraoka-hosp.jp (A.T.); 2 Department of Neurosurgery, The University of Tokyo Hospital, Tokyo 113-8655, Japan; miyawaki-tky@umin.ac.jp (S.M.); SHIN-NSU@h.u-tokyo.ac.jp (M.S.); nsaito-tky@umin.net (N.S.); 3 Diabetes and Metabolism Information Center, Research Institute, National Center for Global Health and Medicine, Tokyo 162-8655, Japan; takehiro.sugiyama@gmail.com; 4 Department of Health Services Research, Faculty of Medicine, University of Tsukuba, Ibaraki 305-8575, Japan; 5 Department of Nephrology, Teraoka Memorial Hospital, Hiroshima 729-3103, Japan; info@teraoka-hosp.jp

**Keywords:** dialysis, stroke, diabetes mellitus, diabetic nephropathy, uremic toxin

## Abstract

The stroke incidence in hemodialysis (HD) patients is high, but the associated factors remain largely unknown. This study aimed to analyze stroke incidence in HD patients and changes in risk factors. Data of 291 patients were retrospectively analyzed. The cumulative stroke incidences were 21.6% at 10 years and 31.5% at 20. Diabetic nephropathy (DN) significantly increased overall stroke (hazard ratio (HR), 2.24; 95% confidence interval (CI), 1.21–4.12; *p* = 0.001) and ischemic stroke (HR, 2.16; 95% CI, 1.00–4.64; *p* = 0.049). Patients treated with online HDF were less likely to have overall stroke (HR, 0.13; 95% CI, 0.03–0.56; *p* = 0.006) and ischemic stroke (HR, 0.08; 95% CI, 0.01–0.60; *p* = 0.014). DN (HR, 1.56; 95% CI, 1.08–2.27; *p* = 0.019) and age >80 years at HD initiation (20–49 years old; HR 0.13, 95% CI, 0.05-0.35, *p* < 0.001 and age 50–79 years; HR 0.42, 95% CI, 0.26–0.66, *p* < 0.001 (reference: age >80 years)) were significantly associated with stroke and/or death events. Over time, stroke risk increased in HD patients, due to the increasing number of DN. Although dialysis technology has advanced over time, these advances could not overcome other risk factors for stroke. Further increase in stroke and mortality due to aging remains a concern.

## 1. Introduction

The increasing number of patients with end-stage renal disease (ESRD) on dialysis and the high incidence of stroke remains a major concern. Compared to the general population, patients with ESRD on dialysis have a 4 to 20 times increased risk of stroke [1,2,3,4,5,6,7]. Reducing stroke incidence is important for prognosis in patients with ESRD on dialysis, considering the high mortality and recurrence rates [8,9].

To date, several risk factors for stroke in patients with ESRD on dialysis have been reported, and include diabetes mellitus (DM), hypertension, atrial fibrillation (Af), old age, anemia, malnutrition, asymmetric dimethylarginine, and elevated C-reactive protein (CRP) [4,6,10,11,12,13,14]. Additionally, we have previously reported that diabetic nephropathy (DN) is a significant risk factor for stroke [9].

Recent improvements in treatment of DM and dialysis technology have resulted in better long-term prognosis in patients with DM or DN. However, the impact of these advances on patients with ESRD on dialysis has not been fully elucidated; the effect on the reduction of stroke incidence should be evaluated.

We performed a single-institution-based retrospective study to analyze changes in the incidence and risk factors of stroke in patients with ESRD on dialysis over time.

## 2. Results

### 2.1. Baseline Characteristics

The baseline characteristics of patients on hemodialysis (HD) in each period are shown in Table 1. The timing of HD introduction was divided into three periods, considering the advances in dialysis techniques in each era: 1979–2009 (early period), 2010–2014 (middle period), and 2015–2020 (late period), and analysis was conducted for each period. A total of 119 HD patients in the early period, 71 in the middle period, and 101 in the late period were included. Patients who had HD initiated in the early period were significantly younger, had a longer observation period, and were more likely to receive anticoagulant therapy, while those in the middle period had a significantly higher incidence of DM and DN. Those in the middle period also had a significantly lower level of CRP and urea reduction ratio (URR). Patients in the late period were more likely to have dyslipidemia (DL) and smoking history, but less likely to have ischemic heart disease and arteriosclerosis obliterans. The differences in the baseline characteristics of patients on HD between with and without online hemodialysis filtration (HDF) are shown in Table S1.

### 2.2. Analysis of Stroke Incidence

A total of 47 patients (16.2%) experienced stroke with an incidence rate of 19.9 per 1000 patient-years. Nineteen (6.5%) were hemorrhagic and 28 (9.6%) were ischemic. Of the 28 patients with ischemic stroke (IS), 18 (6.2%) had lacunar infarction, 4 (1.4%) had atherothrombotic cerebral infarction, and 6 (2.1%) had cerebral embolism (Table 2).

**Figure 1:**
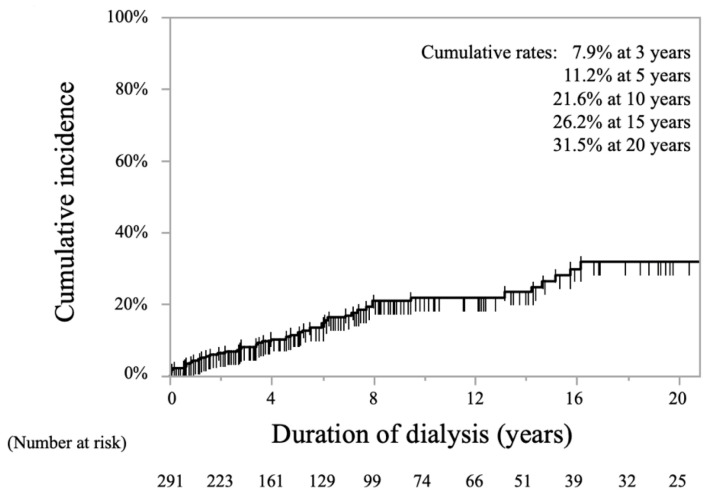
Kaplan-Meier curve showing cumulative incidence rates of stroke in all periods of hemodialysis.

The Kaplan-Meier curve for the cumulative incidence rates of stroke among all patients is shown in Figure 1. The overall cumulative stroke incidence rates were 11.2% at 5 years, 21.6% at 10 years, and 31.5% at 20 years.

**Figure 2:**
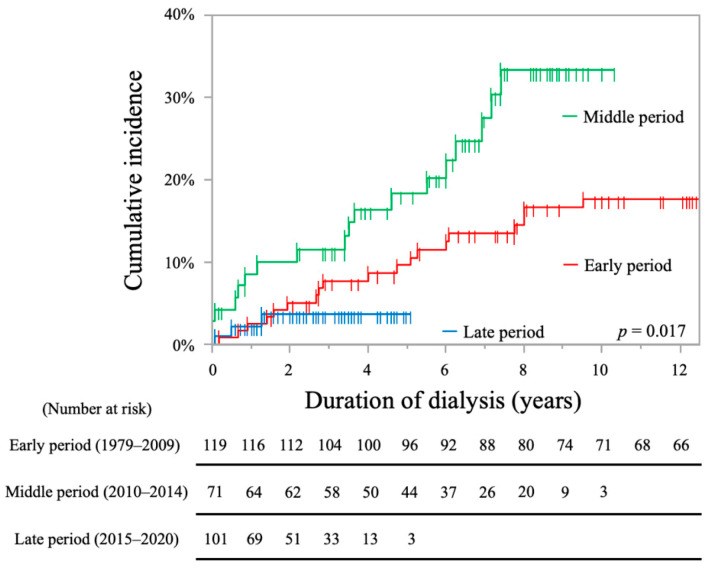
Kaplan-Meier curves showing cumulative incidence rates of stroke in each period.

The cumulative incidence rates of stroke in each period are shown in Figure 2. The cumulative incidence rates in the middle period group were significantly higher than those in the other groups (early period, 15.3/1000 patients-years, 9.6% at 5 years, 17.8% at 10 years; middle period, 40.0/1000 patients-years, 18.3% at 5 years, 33.4% at 10 years; late period, 14.3/1000 patients-years, 3.8% at 5 years; log-rank test, *p* = 0.017).

**Figure 3:**
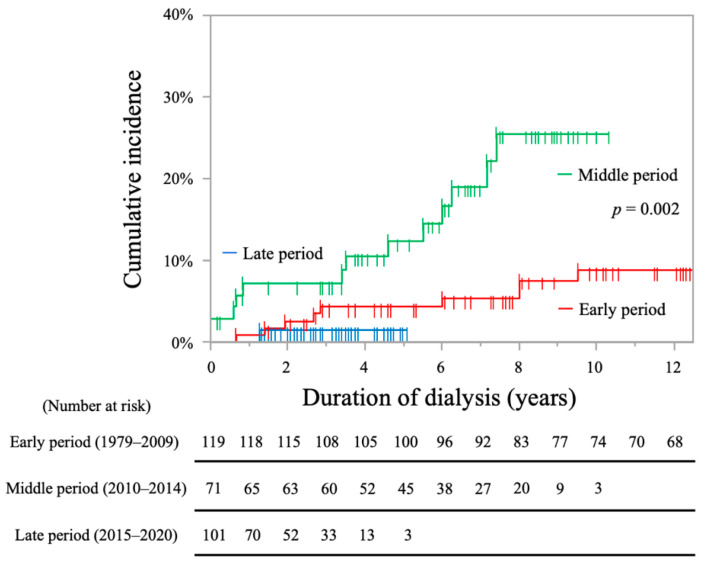
Kaplan-Meier curves showing cumulative incidence rates of ischemic stroke in each period.

The cumulative incidence rates of IS were significantly higher in the middle period group (early period, 4.3% at 5 years, 8.8% at 10 years; middle period, 12.4% at 5 years, 25.6% at 10 years; late period, 1.5% at 5 years; log-rank test, *p* = 0.002, Figure 3).

**Figure 4:**
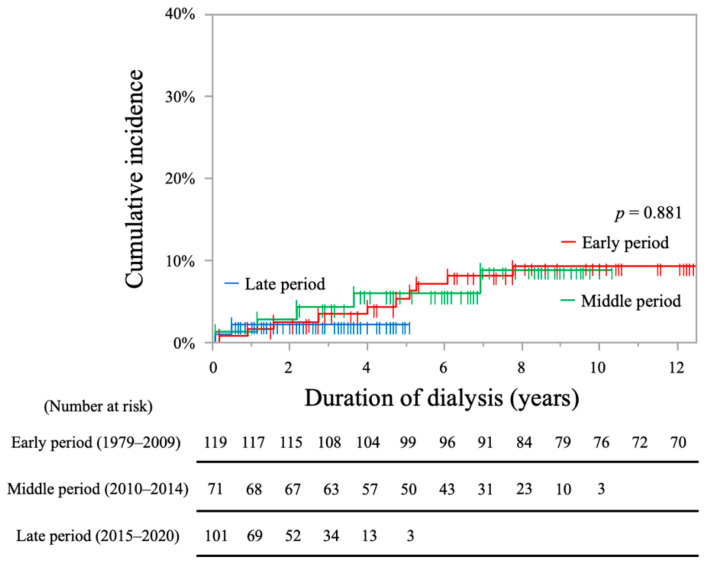
Kaplan-Meier curves showing cumulative incidence rates of hemorrhagic stroke in each period.

The cumulative incidence rates of hemorrhagic stroke (HS) were not significantly different among the three groups (early period, 5.3% at 5 years, 9.3% at 10 years; middle period, 6.0% at 5 years, 8.9% at 10 years; late period 2.2% at 5 years; log-rank test, *p* = 0.881, Figure 4).

**Figure 5:**
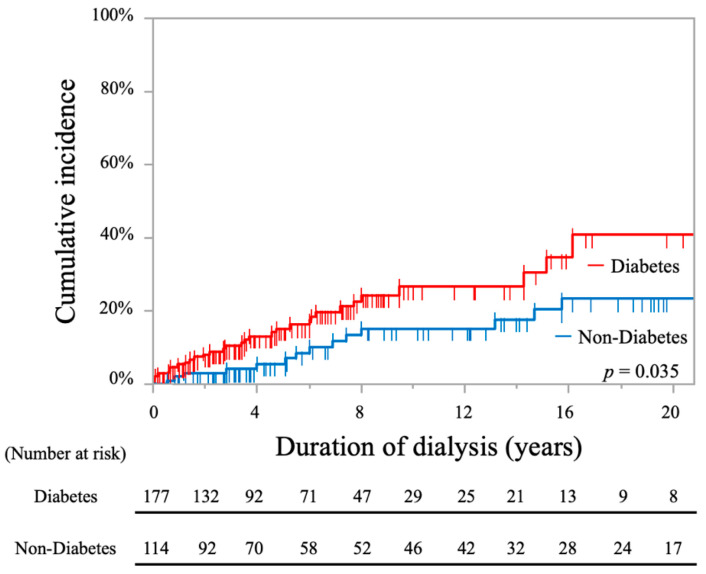
Kaplan-Meier curves showing cumulative incidence rates of stroke in all periods comparing hemodialysis patients with and without diabetes.

The cumulative incidence rates of stroke in patients with DM were significantly higher than those in non-DM patients, as shown by the log-rank test (26.6% at 10 years and 40.7% at 20 years vs. 15.1% at 10 years and 23.2% at 20 years; *p* = 0.035, Figure 5).

**Figure 6:**
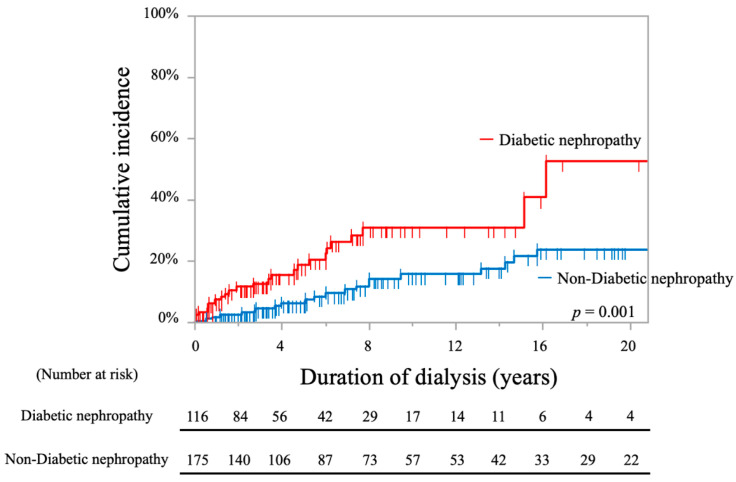
Kaplan-Meier curves showing cumulative incidence rates of stroke in all periods comparing hemodialysis patients with and without diabetic nephropathy.

The cumulative incidence rates of stroke in patients with DN were significantly higher compared to those in non-DN patients, as shown by the log-rank test (30.9% at 10 years and 52.6% at 20 years vs. 15.7% at 10 years and 23.9% at 20 years; *p* = 0.001, Figure 6).

**Figure 7:**
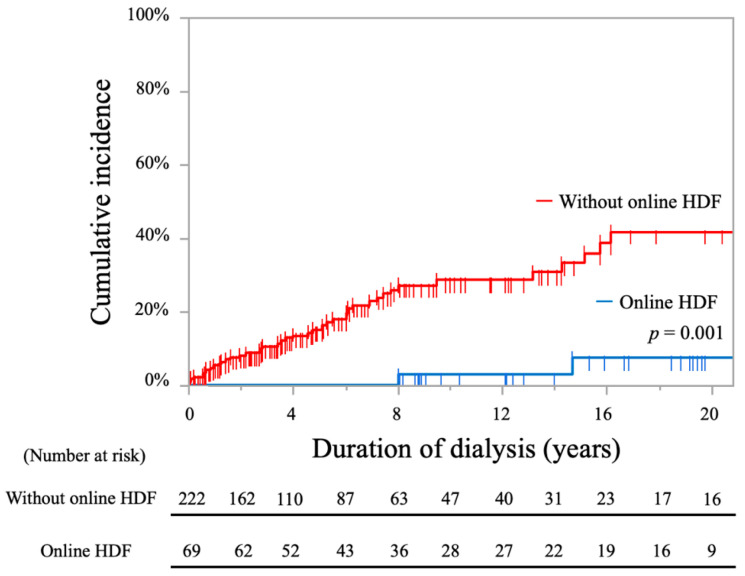
Kaplan-Meier curves showing cumulative incidence rates of stroke in all periods comparing hemodialysis patients with and without online HDF.

The cumulative incidence rates of stroke in patients with online HDF were significantly lower compared to those without, as shown by the log-rank test (2.8% at 10 years and 7.4% at 20 years vs. 28.7% at 10 years and 41.8% at 20 years; *p* = 0.001, Figure 7).

Factors associated with stroke were analyzed using Cox proportional hazards analysis (Table 3). Using bivariate analysis, we found that age at initiation of HD between 20 and 49 years had a significantly lower risk for stroke than initiation at age >80 years (hazard ratio (HR), 0.22; 95% confidence interval (CI), 0.07–0.72; *p* = 0.012). Online HDF showed significantly lower risk for stroke (HR, 0.09; 95% CI, 0.02–0.39; *p* < 0.001). DM (HR, 1.94; 95% CI, 1.04–3.63; *p* = 0.039), DN (HR, 2.54; 95% CI, 1.41–4.58; *p* = 0.002) and URR < 65 (HR, 3.47; 95% CI, 1.81–6.68; *p* < 0.001) were significant risk factors for stroke. Multivariable analysis showed that DN (HR, 2.24; 95% CI, 1.21–4.12; *p* = 0.001), URR < 65 (HR, 3.33; 95% CI, 1.73–6.42; *p* = 0.001) were significant risks for stroke. Online HDF showed significantly lower risk for stroke (HR, 0.13; 95% CI, 0.03–0.56; *p* = 0.006).

Factors associated with IS were also analyzed using Cox proportional hazards analysis (Table 4). Bivariate analysis showed that DN (HR, 2.66; 95% CI, 1.23–5.75; *p* = 0.013) and URR < 65 (HR, 2.42; 95% CI, 1.09–5.37; *p* = 0.030) were significant risk factors for IS, and HD introduction in the early period was associated with significantly lower risk of IS compared to HD initiation in the middle period (HR, 0.37; 95% CI, 0.15–0.91, *p* = 0.030). Online HDF showed a significantly lower risk for IS (HR, 0.08; 95% CI, 0.01–0.59; *p* = 0.013). Meanwhile, multivariable analysis showed that DN was also a significant risk factor (HR, 2.16; 95% CI, 1.00–4.64; *p* = 0.049), and that HD introduction in the early period was associated with significantly lower risk than initiation in the middle period (HR 0.31; 95% CI, 0.13–0.75; *p* = 0.010). Online HDF showed a significantly lower risk for IS (HR, 0.08; 95% CI, 0.01–0.60; *p* = 0.014).

**Figure 8:**
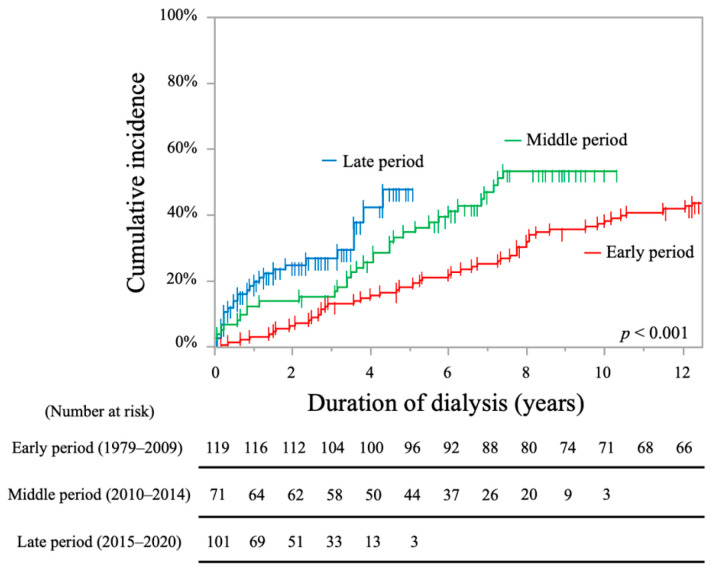
Kaplan-Meier curves showing the cumulative incidence rates of stroke and/or death events in each period.

Additionally, the cumulative incidence rates of stroke and/or death events in each period are shown in Figure 8. The cumulative incidence rates in the late period group were significantly higher than that in the other groups (early period, 18.5% at 5 years, 38.4% at 10 years; middle period, 35.0% at 5 years, 53.3% at 10 years; late period, 47.8% at 5 years; log-rank test, *p* < 0.001).

Factors associated with stroke and/or death events were also analyzed using Cox proportional hazards analysis (Table 5). Bivariate analysis showed that age at initiation of HD between 20 and 49 years had significantly lower risk for stroke and/or death events than initiation at age >80 years (HR, 0.12; 95% CI, 0.05–0.29; *p* < 0.001). DM (HR, 1.82; 95% CI, 1.26–2.63; *p* = 0.001), DN (HR, 1.67; 95% CI, 1.17–2.38; *p* = 0.005) and HD introduction in late period (HR, 2.41; 95% CI, 1.48–3.93, *p* < 0.001), CRP > 0.3 (HR, 2.55; 95% CI, 1.80–3.62; *p* < 0.001), Kt/V (K dialyzer clearance of urea, t dialysis time, V volume of distribution of urea) <1.2 (HR, 1.97; 95% CI, 1.39–2.79, *p* < 0.001) and URR < 65 (HR, 2.61; 95% CI, 1.80–3.78, *p* < 0.001) were significant risk factors for stroke and/or death events. HD introduction in the early period (HR, 0.46; 95% CI, 0.31–0.69, *p* < 0.001) was associated with significantly lower risk for stroke and/or death events compared with HD introduction in the middle period. Online HDF also showed a significantly lower risk for stroke and/or death events (HR, 0.12; 95% CI, 0.06–0.26; *p* < 0.001). Multivariable analysis showed that age at initiation of HD between 20 and 49 years (HR, 0.13; 95% CI, 0.05–0.35; *p* < 0.001) and 50 and 70 years (HR, 0.42; 95% CI, 0.26–0.66; *p* = 0.001) were significantly associated with a lower risk for stroke and/or death events than over 80 years. DN (HR, 1.56; 95% CI, 1.08–2.27; *p* = 0.019), HD introduction in late period (HR, 1.84; 95% CI, 1.06–3.21, *p* = 0.031), CRP > 0.3 (HR, 1.93; 95% CI, 1.35–2.76; *p* = 0.001) and URR < 65 (HR, 2.29; 95% CI, 1.57–3.32, *p* < 0.001) were also significant risk factors for stroke and/or death events. Online HDF was also a significantly lower risk for stroke and/or death events (HR, 0.20; 95% CI, 0.09–0.44; *p* < 0.001).

## 3. Discussion

First, we evaluated the conventional stroke risks such as DM and DN, which can be prevented or improved in advance, and the incidence of stroke in HD patients in the long-term period. Kaplan-Meier analysis showed that IS increased significantly in the middle period when DM and DN also increased. Multivariable analysis also showed that DN and the middle period were significantly associated with IS. Similarly, Kaplan-Meier analysis showed that the entire number of stroke incidences were higher in the middle, early, and late periods, in this order, which may be due to increased incidence of IS. Multivariable analysis showed that DN was a significant risk factor for overall stroke. Furthermore, Kaplan-Meier analysis showed that the incidence of stroke and/or death was higher in the late, middle, and early periods, in this order. The multivariable results showed that older age and DN were significantly associated with stroke and/or death, which may be due to the effects of increased mortality in the late period when the population was older. DN remains the leading cause of dialysis initiation in both Japan and the United States, but the increasing trend has slowed down [15,16,17]. Previous studies have reported that early detection and effective intervention of DM, as well as improved chronic kidney disease management in patients with DM, may contribute to preventing the transition to severe DM and DN and reducing stroke [9,17]. The present study also showed that the ratios of DM and DN have decreased in the late period, and that the incidence of stroke, especially ischemic stroke, has been controlled.

Second, we also evaluated uremic toxins, included in dialysis-related factors, which need to be essentially recognized for the prevention of stroke. We analyzed the relationship between stroke and several laboratory parameters such as CRP, intact parathyroid hormone (PTH), Kt/V, and URR as a measure of low molecular toxins removal by the HD. Inadequate URR control (<65%) was significantly associated with overall and ischemic stroke, stroke, and/or death events. To the best of our knowledge, there have been no reports describing an association between URR and stroke. The responsiveness of ESRD patients to HD at the initiation of dialysis treatment might reflect future stroke.

Furthermore, we analyzed the effect of online HDF on stroke prevention, which can better remove medium molecular weight toxins. The advances in HD techniques have been impressive in recent years. Following the national guidelines in Japan and quality standards for the use of dialyzers [18], we achieved sterile and non-pyrogenic (endotoxin-free) dialysate status since 2010. Changes in insurance coverage of dialysis in Japan has also promoted the introduction of online HDF, and currently half of the dialysis machines in our hospital are used in the online HDF mode, which has been reported to reduce CRP and pro-inflammatory cytokine interleukin 6 [19,20]. In fact, in this study, online HDF contributed to the reduction of stroke onset in HD. The introduction of online HDF and the use of high-purity dialysate to prevent endotoxin entry and elimination of inflammatory proteins have been shown to contribute towards preventing chronic inflammation and reduction of stroke [21,22].

Finally, we give thought to stroke incidence in each period, considering the advances in HD techniques. Although dialysis technology has definitely advanced over time, the incidence of stroke has increased from the early to the middle period, suggesting that advancement in technology may not have sufficiently suppressed stroke incidence. These technological advances could not overcome other risk factors for stroke. For example, increase in DM and DN, and the aging population, might have had an impact beyond these advances; there may also be differences in background factors that were not adjusted for in this study. Our analysis of the incidence of stroke and/or death events showed that the events were highest in the late period when HD techniques would be expected to be most advanced. If the technological advances were overwhelming, the incidence of the events should have decreased. Multivariable analysis weakened this tendency, but the direction of association remained unchanged. Improvements in HD technology are still desirable since several factors, such as population aging, are still increasing dialysis patients’ mortality. Furthermore, since this observational study did not compare each treatment effect, further analyses are needed to determine individual treatment advances.

This study had several limitations. First, this study was based in a single institute with a relatively small number of patients. Therefore, we cannot exclude the possibility of patient selection bias. The trends shown in this study need to be confirmed in well-designed prospective studies. Second, not all previously reported confounding factors were adjusted for. Further studies based on multi-institutional cohorts are required. Third, we compared stroke incidence over three time periods, determined by advances in HD technology. Strictly, these advances need to be treated as time-varying covariates, and the incidence rates for exposure of each advancement should then be compared, although this analysis was limited in our study by the small number of patients included. Fourth, when calculating stroke incidence by subtypes, it is desirable to analyze the stroke subtypes as competing risks.

## 4. Conclusions

This study analyzed the high incidence of stroke and the risk factors in patients on dialysis with long-term follow-up. DN and inadequate URR control significantly increased overall stroke, but online HDF reduced overall stroke. Age >80 years at HD initiation were significantly associated with stroke and/or death events. Although dialysis technology has advanced over time, these advances are not enough to overcome other risk factors for stroke. Further increase in stroke and mortality due to aging remains a concern.

## 5. Materials and Methods

### 5.1. Study Population

All patients were initiating and continuing HD as ESRD renal replacement therapy at Teraoka Memorial Hospital dialysis center between April 1979 and August 2020 were considered for this study. The exclusion criteria were as follows: (a) patients who received HD for acute kidney injury, (b) patients who experienced peritoneal dialysis, and (c) patients who underwent renal transplant surgery before or after initiating HD. This cohort included the 195 patients in our previously reported article [9], but the number of patients increased to 291 cases, and the observation period was extended from a mean of 4.4 to 8.1 years. The present study was newly analyzed for a completely different research question. Considering the retrospective nature of the present study, not all patients were required to provide informed consent since the analysis used anonymous clinical data obtained after each patient had provided written consent for treatment. We also applied the opt-out method to obtain consent on this study’s participation in Teraoka Memorial Hospital’s website. The Institutional Review Board approved disclosure on the website.

### 5.2. HD Procedures and Management

The indication and regimen of HD was based on the clinical practice guidelines of the Japanese Society of Dialysis Medicine [23]. In principle, patients underwent 3–5 h of HD three times per week. Our therapeutic improvements in HD in each era are described below. In the early period, to reduce the risk of cerebral hemorrhage during HD, low-molecular weight anticoagulant heparin was introduced in 1997. Currently, approximately 70% of patients are treated with low-molecular weight heparin, and the remaining 30% with sodium heparin. For patients with a bleeding tendency (with bleeding complications), nafamostat mesylate is temporarily administered. Additionally, central dialysis fluid delivery system was introduced in 1999, which is commonly used in Japan. In the middle period, to reduce the risk of stroke due to vascular inflammation, we promoted the purification of dialysis fluid and achieved aseptic and fever inducing-free material (no endotoxins) in all units since 2010. Online HDF was also introduced in 2012, and the calcium concentration of the dialysate solution was changed from 2.5 to 2.75 mEq/L in 2015, considering the increasing stroke risk due to hypocalcemia. In the late period, HD is performed using all these advanced techniques. The online HDF was performed using high-flux dialyzers based on polysulfone (NVF-13H, NVF-15H, NVF-21H and NVF-26P [Toray Medical Co., Inc., Tokyo, Japan]; ABH-21P and ABH-22PA [Asahi Kasei Medical Co., Inc., Tokyo, Japan]) and poly-ethersulfone (MFX-09M eco, MFX-11M eco, MFX-11E eco, MFX-13E eco, MFX-15E eco, MFX-17E eco, MFX-15S and MFX-21S eco [NIPRO CORPORATION, Inc., Osaka, Japan]). The treatment duration ranged between 240 and 270 min with the quantity of dialysate flow rate 500 mL/min, and the total volume of substitution fluid per session was from 8 to 40 L in predilution mode.

### 5.3. Diagnosis of Stroke and Renal Diseases

All symptomatic events of stroke were diagnosed using computed tomography or magnetic resonance imaging. The diagnosis of stroke was divided into IS and HS. HS included intracerebral hemorrhage and subarachnoid hemorrhage, without traumatic changes.

The diagnosis of renal diseases was based on the clinical context, including personal and family history, social and environmental factors, medications, physical examination, laboratory measures, imaging, and pathologic diagnosis [24]. Histological diagnosis using kidney biopsy was performed in cases that required pathological confirmation, given its invasiveness. The diagnosis of renal disease was classified into five subtypes: DN, nephrosclerosis, glomerulonephritis, polycystic kidney disease, and other nephropathies.

### 5.4. Biochemical Determinations

We evaluated CRP, PTH, URR, and Kt/V as uremic toxins in the blood samples collected at the time of initiation of dialysis. Samples were measured with an auto-analyzer using standard procedures in the Teraoka Memorial Hospital’s laboratory, except for PTH. PTH was measured by the dedicated subcontractor (BML, Inc., Tokyo, Japan). The borderline for CRP was 0.3 mg/dL and for PTH was 240 pg/mL as intact-PTH. URR was calculated from pre- and post-dialysis blood urea nitrogen. Kt/V was calculated using the model in the statistical survey of Japanese Society for Dialysis Therapy. In accordance with the guidelines, the borderline value for URR was 65% and for Kt/V was 1.2 [23].

### 5.5. Statistical Analysis

The baseline characteristics of all patients were summarized and compared among each period. The chi-square test was used for categorical variables and the Mann-Whitney U test for continuous variables. The incidence of stroke was calculated using Kaplan-Meier analysis. For all incidences of stroke, IS and HS were compared using the log-rank test. The time of initiation of HD was defined as time 0. The time to the first stroke event was defined as the interval between time 0 and the month in which the first stroke occurred. Patients without stroke were censored at the final follow-up date or date of death. Factors potentially related to stroke were examined using bivariate and multivariable Cox proportional analysis. Considering the number of patients with stroke, the number of independent variables in the multivariable Cox proportional hazard model should be limited to approximately five. Based on previous studies and results from bivariate analyses in the present study, we included age at initiation of HD, DN, online HDF and URR for stroke, and DN, period of HD introduction, and online HDF and URR for IS in the model. Age at initiation of HD was entered into models as between 20–49 years, 50–70 years, and ≥ 80 years. DM, DL, DN, and Af were also present in patients at the time of stroke onset. Lastly, the incidence rates of stroke and/or death were calculated using Kaplan-Meier analysis and compared using the log-rank test. Considering fatal events as a semi-competing risk for stroke, factors potentially associated with stroke and/or death were also examined using bivariate and multivariable Cox proportional analysis. We also included age at initiation of HD, DN, period of HD introduction, online HDF, CRP, and URR in the multivariable model. All analyses were performed using JMP Pro version 15.0.0 (SAS Institute Inc., Cary, NC). *p* < 0.05 was considered statistically significant.

## Figures and Tables

**Table 1:** Clinical characteristics of patients in each period of dialysis initiation.

	All	1979–2009	2010–2014	2015–2020	*p* Value
Number of patients, *n*	291	119	71	101	/
Age at initiation of dialysis					
Mean ± SD	65.6 ± 14.4	59.1 ± 13.9	70.4 ± 11.4	69.8 ± 14.2	<0.001 *
Range (years)	21–95	21–90	43–93	33–95	
Follow-up period (years)					
Mean ± SD	8.1 ± 7.8	14.3 ± 8.5	6.3 ± 2.5	2.1 ± 1.5	<0.001 *
Range (years)	0.1–38.3	0.3–38.3	0.2–10.3	0.1–5.1	
Male, *n* (%)	187 (64.3%)	68 (57.1%)	46 (64.8%)	73 (72.3%)	0.065
Underlying diseases, *n* (%)					
Hypertension	283 (97.3%)	118 (99.2%)	69 (97.2%)	96 (95.0%)	0.178
Diabetes	177 (60.8%)	65 (54.6%)	52 (73.2%)	60 (59.4%)	0.037 *
Dyslipidemia	114 (39.2%)	34 (28.6%)	28 (39.4%)	52 (51.5%)	0.002 *
Ischemic heart diseases	132 (45.4%)	63 (52.9%)	38 (53.5%)	31 (30.7%)	0.001 *
Arteriosclerosis obliterans	92 (31.6%)	42 (35.3%)	28 (39.4%)	22 (21.8%)	0.026 *
Atrial fibrillation	31 (10.7%)	47 (14.3%)	6 (8.5%)	8 (7.9%)	0.252
Smoking, *n* (%)	110 (37.8%)	32 (26.9%)	26 (36.6%)	52 (51.5%)	0.001 *
Alcohol, *n* (%)	51 (17.5%)	16 (13.5%)	12 (16.9%)	23 (22.8%)	0.191
Online HDF, *n* (%)	69 (23.1%)	29 (24.4%)	18 (25.4%)	22 (21.8%)	0.842
Antiplatelet therapy, *n* (%)	148 (50.9%)	62 (52.1%)	40 (56.3%)	46 (45.5%)	0.356
Anticoagulant therapy, *n* (%)	35 (12.0%)	21 (17.7%)	7 (9.9%)	7 (6.9%)	0.042 *
Primary renal diagnosis of the patients, *n* (%)					
Diabetic nephropathy	116 (39.9%)	40 (33.6%)	33 (46.5%)	43 (42.6%)	0.001 *
Nephrosclerosis	75 (25.8%)	22 (18.5%)	26 (36.6%)	27 (26.7%)	
Glomerulonephritis	44 (15.1%)	29 (24.4%)	6 (8.5%)	9 (8.9%)	
Polycystic kidney disease	11 (3.8%)	5 (4.2%)	2 (2.8%)	4 (4.0%)	
Others	45 (15.5%)	23 (19.3%)	4 (5.6%)	18 (17.8%)	
Laboratory parameters					
CRP (mg/dL)					
Mean ± SD	0.97 ± 3.27	0.92 ± 2.93	0.59 ± 1.42	1.29 ± 4.38	0.001 *
Range (mg/dL)	0.00–30.98	0.00–26.1	0.00–10.1	0.00–30.98	
PTH (pg/mL)					
Mean ± SD	183 ± 177	191 ± 179	187 ± 227	172 ± 126	0.965
Range (pg/mL)	5–1842	6–1260	5–1842	7–724	
Kt/V					
Mean ± SD	1.27 ± 0.33	1.30 ± 0.33	1.20 ± 0.31	1.29 ± 0.33	0.061
Range	0.48–2.21	0.48–2.21	0.59–1.82	0.51–2.16	
URR (%)					
Mean ± SD	62.9 ± 10.6	63.5 ± 11.3	59.6 ± 11.1	64.4 ± 8.8	0.011 *
Range (%)	26.7–93.9	26.7–93.9	32.6–77.8	36.1–82.5	

*n*, number; SD, standard deviation; HDF, hemodialysis filtration; CRP, C-reactive protein; PTH, parathyroid hormone; Kt/V, K dialyzer clearance of urea, t dialysis time, V volume of distribution of urea; URR, urea reduction ratio. * Values of *p* < 0.05 are considered statistically significant.

**Table 2:** Subtype of stroke in each period.

Period	Patient-Years	All Stroke		Hemorrhagic	Ischemic			
		*n* (%)	IR	*n* (%)	All *n* (%)	Lacunar *n* (%)	Atherothrombotic *n* (%)	Embolism *n* (%)
Entire (*n* = 291)	2360	47 (16.2)	19.9	19 (6.5)	28 (9.6)	18 (6.2)	4 (1.4)	6 (2.1)
Early (*n* = 119)	1700	26 (21.9)	15.3	12 (10.1)	14 (11.8)	11 (9.2)	2 (1.7)	1 (0.8)
Middle (*n* = 71)	450	18 (25.4)	40.0	5 (7.0)	13 (18.3)	7 (9.9)	2 (2.8)	4 (5.6)
Late (*n* = 101)	210	3 (3.0)	14.3	2 (2.0)	1 (1.0)	0 (0.0)	0 (0.0)	1 (1.0)

*n*, number; IR, incidence rates (1000 patient-years).

**Table 3:** Results of bivariate and multivariable analyses of factors associated with stroke in patients on hemodialysis.

	Bivariate		Multivariable	
	HR (95% CI)	*p* Value	HR (95% CI)	*p* Value
Age at initiation of HD (ref: 80 years old or over)				
20–49 years-old	0.22 (0.07–0.72) ^†^	0.012 *	0.42 (0.10–1.85) ^†^	0.253
50–79 years-old	1.90 (0.91–3.96) ^†^	0.088	0.71 (0.28–1.77) ^†^	0.464
Hypertension	1.9 × 10^8^ (0–N/A)	0.999		
Diabetes (ref: non-diabetes)	1.94 (1.04–3.63)	0.039 *	/	/
Dyslipidemia (ref: non-dyslipidemia)	1.03 (0.56–1.89)	0.919	/	/
Diabetic nephropathy (ref: non-diabetic nephropathy)	2.54 (1.41–4.58)	0.002 *	2.24 (1.21–4.12)	0.001 *
Period (ref: middle period)				
Early period	0.60 (0.31–1.15) ^‡^	0.598	/	/
Late period	0.42 (0.12–1.43) ^‡^	0.167	/	/
Online HDF (ref: without online HDF)	0.09 (0.02–0.39)	<0.001 *	0.13 (0.03–0.56)	0.006 *
Atrial fibrillation (ref: non-atrial fibrillation)	1.33 (0.59–2.96)	0.491	/	/
Laboratory parameters				
CRP > 0.3 (ref: CRP ≤ 0.3)	1.62 (0.88–2.98)	0.122	/	/
PTH > 240 (ref: PTH ≤ 240)	1.65 (0.89–3.07)	0.110	/	/
Kt/V < 1.2 (ref: Kt/V ≥ 1.2)	1.66 (0.93–2.96)	0.088	/	/
URR < 65 (ref: URR ≥ 65)	3.47 (1.81–6.68)	<0.001 *	3.33 (1.73–6.42)	0.001 *

* Values of *p* < 0.05 are considered statistically significant. ^†^ The values of HR and 95% CI are based on age at initiation of HD (≥80 years of age). ^‡^ The values of HR and 95% CI are based on middle period. HD, hemodialysis; HR, hazard ratio; CI, confidence interval; ref, reference; N/A, not available; HDF, hemodialysis filtration; CRP, C-reactive protein; PTH, parathyroid hormone; Kt/V, K dialyzer clearance of urea, t dialysis time, V volume of distribution of urea; URR, urea reduction ratio.

**Table 4:** Bivariate and multivariable analyses of factors associated with ischemic stroke in patients on hemodialysis.

	Bivariate		Multivariable	
	HR (95% CI)	*p* Value	HR (95% CI)	*p* Value
Age at initiation of HD (ref: 80 years old or over)				
20–49 years-old	0.23 (0.05–1.01) ^†^	0.051	/	/
50–79 years-old	2.11 (0.78–5.63) ^†^	0.137	/	/
Hypertension	1.9 × 10^8^ (0–N/A)	0.999		
Diabetes (ref: non-diabetes)	1.99 (0.88–4.51)	0.097	/	/
Dyslipidemia (ref: non-dyslipidemia)	0.79 (0.35–1.81)	0.585	/	/
Diabetic nephropathy (ref: non-diabetic nephropathy)	2.66 (1.23–5.75)	0.013 *	2.16 (1.00–4.64)	0.049 *
Period (ref: middle period)				
Early period	0.37 (0.15–0.91) ^‡^	0.030 *	0.31 (0.13–0.75) ^‡^	0.010 *
Late period	0.25 (0.03–1.92) ^‡^	0.248	0.16 (0.02–1.29) ^‡^	0.085
Online HDF (ref: without online HDF)	0.08 (0.01–0.59)	0.013 *	0.08 (0.01–0.60)	0.014 *
Atrial fibrillation (ref: non-atrial fibrillation)	1.71 (0.65–4.51)	0.279	/	/
Laboratory parameters				
CRP > 0.3 (ref: CRP ≤ 0.3)	2.13 (0.99–4.58)	0.054	/	/
PTH > 240 (ref: PTH ≤ 240)	1.57 (0.71–3.49)	0.269	/	/
Kt/V < 1.2 (ref: Kt/V ≥ 1.2)	2.00 (0.94–4.27)	0.073	/	/
URR < 65 (ref: URR ≥ 65)	2.42 (1.09–5.37)	0.030 *	2.00 (0.88–4.52)	0.096

* Values of *p* < 0.05 are considered statistically significant. ^†^ The values of HR and 95% CI are based on age at initiation of HD (≥80 years of age). ^‡^ The values of HR and 95% CI are based on middle period. HD, hemodialysis; HR, hazard ratio; CI, confidence interval; ref, reference; N/A, not available; HDF, hemodialysis filtration; CRP, C-reactive protein; PTH, parathyroid hormone; Kt/V, K dialyzer clearance of urea, t dialysis time, V volume of distribution of urea; URR, urea reduction ratio.

**Table 5:** Bivariate and multivariable analyses of factors associated with stroke and/or death in patients on hemodialysis.

	Bivariate		Multivariable	
	HR (95% CI)	*p* Value	HR (95% CI)	*p* Value
Age at initiation of HD (ref: 80 years old or over)				
20–49 years-old	0.12 (0.05–0.29) ^†^	<0.001 *	0.13 (0.05–0.35) ^†^	<0.001 *
50–79 years-old	1.11 (0.76–1.63) ^†^	0.580	0.42 (0.26–0.66) ^†^	0.001 *
Hypertension	0.42 (0.18–0.96)	0.039 *		
Diabetes (ref: non-diabetes)	1.82 (1.26–2.63)	0.001 *	/	/
Dyslipidemia (ref: non-dyslipidemia)	0.91 (0.63–1.32)	0.622	/	/
Diabetic nephropathy (ref: non-diabetic nephropathy)	1.67 (1.17–2.38)	0.005 *	1.56 (1.08–2.27)	0.019 *
Period (ref: middle period)				
Early period	0.46 (0.31–0.69) ^‡^	<0.001 *	0.69 (0.43–1.10) ^‡^	0.125
Late period	2.41 (1.48–3.93) ^‡^	<0.001 *	1.84 (1.06–3.21) ^‡^	0.031 *
Online HDF (ref: without online HDF)	0.12 (0.06–0.26)	<0.001 *	0.20 (0.09–0.44)	<0.001 *
Atrial fibrillation (ref: non-atrial fibrillation)	1.27 (0.78–2.06)	0.341	/	/
Laboratory parameters				
CRP > 0.3 (ref: CRP ≤ 0.3)	2.55 (1.80–3.62)	<0.001 *	1.93 (1.35–2.76)	0.001 *
PTH > 240 (ref: PTH ≤ 240)	0.85 (0.55–1.30)	0.451	/	/
Kt/V < 1.2 (ref: Kt/V ≥ 1.2)	1.97 (1.39–2.79)	<0.001 *	/	/
URR < 65 (ref: URR ≥ 65)	2.61 (1.80–3.78)	<0.001 *	2.29 (1.57–3.32)	<0.001 *

* Values of *p* < 0.05 are considered statistically significant. ^†^ The values of HR and 95% CI are based on age at initiation of HD (≥80 years of age). ^‡^ The values of HR and 95% CI are based on middle period. HD, hemodialysis; HR, hazard ratio; CI, confidence interval; ref, reference; HDF, hemodialysis filtration; CRP, C-reactive protein; PTH, parathyroid hormone; Kt/V, K dialyzer clearance of urea, t dialysis time, V volume of distribution of urea; URR, urea reduction ratio.

## Data Availability

Data collected for the study, including individual participant data, are not available because of the no patient approval. Information about the analytic method will be available from the corresponding author upon reasonable request.

## Supplementary Materials

The following are available online at https://www.mdpi.com/2072-6651/13/5/350/s1, Table S1: Clinical characteristics of patients in each period of dialysis initiation.
