# Supplementary material for: Changes in the Risk of Stroke in Dialysis Patients: A Retrospective Analysis over the Last 40 Years
Source: Toxins (Basel). 2021 May 13;13(5):350. doi: 10.3390/toxins13050350 (PMC8170903; doi:10.3390/toxins13050350)
Supplement: Supplementary file 1 [file toxins-13-00350-s001.zip › toxins-1211184-supp final.pdf]

Changes in the Risk of Stroke in Dialysis Patients: A Retrospective Analysis over the Last 40 Years

Toshiya Aono, Yuki Shinya, Satoru Miyawaki, Takehiro Sugiyama, Isao Kumagai, Atsumi Takenobu, Masahiro Shin, Nabuhito Saito and Akira Teraoka

Table S1. Clinical characteristics of patients in each period of dialysis initiation.

	HD	Online HDF	*p* Value
Number of patients, n	222	69	/
Age at initiation of dialysis			
Mean ± SD	68.3 ± 13.1	56.6 ± 15.0	<0.001*
Range (years)	27–95	21–88	
Follow-up period (years)			
Mean ± SD	7.4 ± 7.5	10.5 ± 8.4	0.002*
Range (years)	0.1–38.3	0.3–36.2	
Male, n (%)	139 (62.6%)	48 (69.6%)	0.293
Underlying diseases, n (%)			
Hypertension	214 (96.4%)	69 (100.0%)	0.110
Diabetes	145 (65.3%)	32 (46.4%)	0.005*
Dyslipidemia	86 (38.7%)	28 (40.6%)	0.784
Ischemic heart diseases	109 (49.1%)	23 (33.3%)	0.022*
Arteriosclerosis obliterans	70 (31.5%)	22 (31.9%)	0.956
Atrial fibrillation	27 (30.3%)	4 (36.4%)	0.684
Smoking, n (%)	74 (33.3%)	36 (52.2%)	0.005*
Alcohol, n (%)	33 (14.9%)	18 (26.1%)	0.032*
Antiplatelet therapy, n (%)	117 (52.7%)	31 (44.9%)	0.259
Anticoagulant therapy, n (%)	30 (13.5%)	5 (7.3%)	0.162
Primary renal diagnosis of the patients, n (%)			
Diabetic nephropathy	94 (42.3%)	22 (31.9%)	
Nephrosclerosis	64 (28.8%)	11 (15.9%)	
Glomerulonephritis	32 (14.4%)	12 (17.4%)	<0.001*
Polycystic kidney disease	9 (4.1%)	2 (2.9%)	
Others	23 (10.4%)	22 (31.9%)	
Laboratory parameters			
CRP (mg/dL)			
Mean ± SD	1.16 ± 3.66	0.35 ± 1.22	0.007*
Range (mg/dL	0.00–30.98	0.00–9.90	
PTH (pg/mL)			
Mean ± SD	185 ± 191	179 ± 120	0.464
Range (pg/mL)	5–1842	10–537	
Kt/V			
Mean ± SD	1.26 ± 0.33	1.32 ± 0.31	0.208
Range	0.48–2.21	0.52–2.16	
URR (%)			

Mean ± SD	62.0 ± 10.8	65.7 ± 9.3	0.026*
Range (%)	26.7–84.3	35.6–93.9	

n, number; SD, standard deviation; HDF, hemodialysis filtration; CRP, C-reactive protein; PTH, parathyroid hormone; Kt/V, K dialyzer clearance of urea, t dialysis time, V volume of distribution of urea; URR, urea reduction ratio.

* Values of $p < 0.05$ are considered statistically significant.
